# A comparison between quantum chemistry and quantum Monte Carlo techniques for the adsorption of water on the (001) LiH surface

**DOI:** 10.1063/1.4984048

**Published:** 2017-05-26

**Authors:** Theodoros Tsatsoulis, Felix Hummel, Denis Usvyat, Martin Schütz, George H. Booth, Simon S. Binnie, Michael J. Gillan, Dario Alfè, Angelos Michaelides, Andreas Grüneis

**Affiliations:** 1 Max Planck Institute for Solid State Research, Heisenbergstraße 1, D-70569 Stuttgart, Germany; 2 Institut für Chemie, Humboldt-Universität zu Berlin, Brook-Taylor-St. 2, D-12489 Berlin, Germany; 3 Department of Physics, King’s College London, Strand, London WC2R 2LS, United Kingdom; 4 London Centre for Nanotechnology, University College London, Gordon St., London WC1H 0AH, United Kingdom; 5 Thomas Young Centre, University College London, London WC1H 0AH, United Kingdom; 6 Department of Physics and Astronomy, University College London, London WC1E 6BT, United Kingdom; 7 Department of Earth Sciences, University College London, London WC1E 6BT, United Kingdom; 8 Department Chemie, Technische Universität München, Lichtenbergstraße 4, D-85747 Garching, Germany

## Abstract

We present a comprehensive benchmark study of the adsorption energy of a single water molecule on the (001) LiH surface using periodic coupled cluster and quantum Monte Carlo theories. We benchmark and compare different implementations of quantum chemical wave function based theories in order to verify the reliability of the predicted adsorption energies and the employed approximations. Furthermore we compare the predicted adsorption energies to those obtained employing widely used van der Waals density-functionals. Our findings show that quantum chemical approaches are becoming a robust and reliable tool for condensed phase electronic structure calculations, providing an additional tool that can also help in potentially improving currently available van der Waals density-functionals.

## I. INTRODUCTION

Kohn–Sham density-functional theory (DFT) is the standard approach for the first-principles description of electronic properties in computational material science and surface chemistry. However, it is becoming clear that the limitations of the employed exchange-correlation (XC) functionals to balance off the numerous competing physical effects give rise to deficiencies in the predictive ability of the approach, generally without any systematic manner to improve upon it. One class of widely studied problems where this is particularly true is the case of molecular adsorption on periodic surfaces. Competing physical effects as well as poorly treated long-range dispersion contributions result in predicted adsorption energies and sites varying strongly with the employed XC functional (see, e.g., Refs. 1–5). This indicates fundamental shortcomings in many semi-local functionals that are difficult to remedy. Long-range dispersive interactions can be accounted for by the addition of pairwise interatomic *C*_6_*R*^−6^ terms to the DFT energy or by non-local functionals.^6–8^ In this work, we will refer to both the van der Waals corrected and van der Waals inclusive DFT methods as van der Waals density-functionals. Theoretically these corrections can be well justified and derived using quantum Drude oscillators that serve as a qualitatively correct model for electrical response properties between molecules and insulating solids. However, most van der Waals corrections also require the introduction of some adjustable parameters such as the cutoff function and cutoff radius at short distances *R* in order to remove the attractive singularity from the *C*_6_*R*^−6^ terms. These parameters can be obtained by optimizing the accuracy of the dispersion corrected functionals for the description of molecular interaction energies in a given test set.

In this work, we consider an *ab initio* description of the true many-body wave function for a molecular adsorption problem. Two contrasting yet complementary approaches which we consider here are those from the field of quantum chemical Fock-space expansions of the wave function^9^ and a stochastic representation from the Diffusion Monte Carlo (DMC) technique.^10^ These wave function based approaches offer a thorough description of quantum many-body effects through a direct treatment of electronic correlation. Such approaches can supplement density-functional-based methods with accurate results.

DMC is a real-space quantum Monte Carlo (QMC) method, where the real-space configurations of all *N*-electrons are sampled stochastically. This stochastic distribution of electrons is evolved toward a sampling of the ground-state distribution of electrons via an imaginary-time propagator, which exponentially filters out the higher-lying eigenfunctions of the Hamiltonian from the distribution. This sampling would be exact if it were not for the “Fermion sign problem,” where the sampling collapses to the lower-energy symmetric distribution of an *N*-particle Bosonic distribution. To avoid this, constraints are imposed whereby the correct antisymmetry is maintained by imposing a hard nodal surface for the sampling which enforces the sign of the sampled configurations. While this alleviates the Fermion sign problem, it introduces a systematic and variational error due to this nodal surface, which in practical applications is generally taken to be the nodal surface of a single Slater determinant. This represents the leading error of a DMC calculation, but it benefits from a number of appealing properties which contrast with the quantum chemical methods, such as a very minor dependence on the basis set, as well as a low-scaling with respect to the system size. DMC techniques are increasingly used to understand molecular adsorption at periodic surfaces.^4,5,11,12^

Quantum chemical methods constitute a hierarchy which starting from the one-particle Hartree–Fock (HF) approximation, allows for a systematic treatment of the quantum many-body effects. The simplest form of such correlated methods is the second-order Møller–Plesset (MP2) perturbation theory. Although MP2 theory provides a fair compromise between efficiency and accuracy, certain effects are not captured accurately enough or at all (e.g. three-body dispersion interactions). For systems where such effects are essential, the accuracy of the MP2 treatment is rather modest. For instance, MP2 is known to notoriously overestimate dispersion driven interactions in strongly polarizable systems.^13–15^ While many-body perturbation theory offers a finite-order approximation to the electronic correlation, coupled-cluster theory provides a compelling framework of infinite-order approximations in the form of an exponential of cluster operators. The coupled-cluster singles and doubles (CCSD) method where the triples are treated in a perturbative way, termed as CCSD(T), achieves chemical accuracy in the description of many molecular properties and is often referred to as the gold standard method.^9^ In recent years, quantum chemical wave function based methods have been increasingly applied to periodic systems with the aim of transferring their proven chemical accuracy in molecular systems to the solid state.^16–26^ However, the computational cost of quantum chemical wave function based methods is a major obstacle for their application to extended systems. The canonical formulation of MP2 theory scales as $O\left(N^{5}\right)$, where *N* is a measure of the system size, whereas CCSD theory scales as $O\left(N^{6}\right)$ and CCSD(T) as $O\left(N^{7}\right)$.

This adverse scaling can in part be attributed to the use of canonical one-electron Bloch orbitals. While canonical orbitals form a convenient basis for correlated calculations since the Fock matrix is then diagonal, they are intrinsically delocalized, rendering it difficult to build in the local character of electronic correlation. In contrast, local correlation schemes^27,28^ exploit the fact that two-point correlations rapidly decay with distance in insulating systems, by restricting excitations to spatially confined regions within localized orbitals. It is possible to therefore reduce the scaling of the canonical quantum chemical methods, in some cases to an asymptotic linear scaling.^29,30^ Several different local approximations exist and represent a highly active field of research. The method of increments relies on a similar local decomposition of the energy contribution and has been applied successfully to covalent large band-gap semiconductors, van der Waals bonded rare-gas or molecular crystals, and molecular adsorption on surfaces.^25,31–39^

In this work, we will consider both local and canonical MP2 approaches in similar basis sets, as well as comparing to both higher-level canonical coupled-cluster and the contrasting DMC technique for the challenging problem of molecular adsorption on a periodic surface. Canonical CCSD theory will be explored within the projector-augmented-wave (PAW) framework, using a plane-wave basis. CCSD(T) theory will be applied in the form of corrections to MP2 with small supercells and basis sets or using finite-clusters. We assess the accuracy of these quantum chemical schemes against the DMC results for water adsorption on the prototypical ionic surface of lithium hydride (LiH). LiH has served as an important benchmark system for several quantum-chemical methods^18,22,23,40–43^ and water adsorption on the (001) LiH surface can, in turn, be a benchmark system for the interaction of molecules with surfaces. The relatively small number of electrons involved allows for an in-depth comparison of different post-mean-field methods.

Details about the structure of the system under consideration are given in Sec. II A. Computational details are presented in Secs. II B–II D for plane-wave, Gaussian basis, and DMC calculations, respectively. Section III summarizes all the results obtained from different methods. Finally, we conclude the paper in Sec. IV.

## II. COMPUTATIONAL DETAILS

### A. H_2_O on LiH geometry

The aim of this work is to compare different high-level theories for the calculation of the adsorption energy of a single water molecule on the (001) LiH surface, keeping the atomic structure of the surface fixed. The adsorption energy is defined as the difference in energy between the non-interacting fragments (water and the LiH surface) and the interacting system (water molecule on LiH),

$$E_{\text{ads}}=E_{{\text{H}}_{2}\text{O}}+E_{\text{LiH}}-E_{{\text{H}}_{2}\text{O}+\text{LiH}}.$$ (1)

**FIG. 1.**
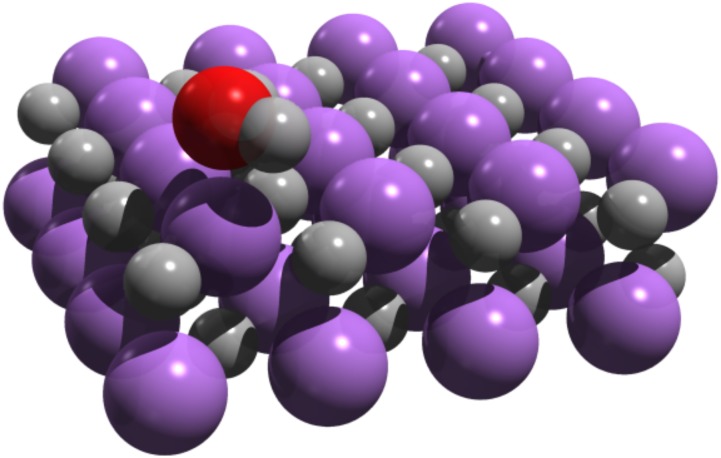
The adsorption geometry of water on a two-layer slab with 64 atoms per cell, representing the (001) LiH surface. The oxygen–surface distance is 2.15 Å, while the water molecule almost retains its equilibrium structure. The geometry was optimized using the PBE functional.

An alternative definition for the adsorption energy is the difference between the energy of the system with the water molecule at its equilibrium position on the surface and that of the system in which the water molecule has been displaced vertically by 10 Å. In both definitions, the molecular structure of the water molecule has been kept the same. The latter definition is used for the DMC calculations since it allows to maximize the possible cancellation of errors.^44^ We stress that since we are primarily interested in benchmarking different electronic-structure methods, zero-point energy contributions or finite temperature effects are neglected. The structure of the surface with the adsorbed molecule has been obtained in the following manner. The Li and H atoms have been kept fixed to their pristine lattice sites with a lattice constant of *a* = 4.084 Å, consistent with the previous studies of the LiH crystal.^19,20,40^ This has the advantage of keeping the geometry consistent when supercells or fragments of different sizes are used in quantum chemical and DMC calculations. The water molecule was relaxed on the LiH (001) surface using the Perdew–Burke–Ernzerhof (PBE) XC functional^45^ and a two-layer slab with the 4 × 4 surface supercell. For these calculations, the vasp code has been employed.^46^ A vacuum gap of 20.5 Å has been employed to ensure that the surface slab does not interact with its periodic image. The relaxed geometry of the water molecule adsorbed on the LiH surface is shown in Fig. 1. The DMC adsorption energy curve obtained by varying the distance between the molecule and the surface agrees well with the oxygen–surface distance of the PBE functional (2.15 Å).^47^ The structural coordinates of Fig. 1 are given in the supplementary material. This geometry is used throughout the paper for all density-functional and correlated calculations. The convergence of the adsorption energy with the number of layers in the slab is explored in Sec. III B.

### B. Plane-wave basis set calculations

The calculations using a plane-wave basis set presented in this work have been performed using the vasp code employing the PAW method alongside with the Γ-point approximation to sample the first Brillouin zone. The kinetic energy cutoff that determines the size of the plane-wave basis set expansion of the one-particle states was set to 500 eV. There are numerous density-functionals that could be considered, of which we have only chosen a small selection. Thus, we assess the accuracy of one of the most widely used functionals, the PBE functional, as well as of several van der Waals functionals. Specifically, dispersion corrections were taken into account following the approach of Grimme *et al.*,^48^ the method of Tkatchenko and Scheffler,^49^ and the van der Waals density-functional (vdW-DF) method proposed by Dion *et al.*,^50–53^ as implemented in vasp. In the former schemes, a correction is added to the DFT total energy after the self-consistent-field (SCF) cycle is converged, whereas the latter scheme is a non-local correlation functional that approximately accounts for dispersion interactions. In all calculations, all electronic states of the H and Li atoms were treated as valence states, whereas the 1*s*^2^ states of the O atom were kept frozen. Supercells of different sizes were used to model the LiH surface, containing 32, 64, and 128 atoms.

In the current paper, we employ pseudized Gaussian-type orbitals (PGTOs) expanded in a plane-wave basis set to span the virtual orbital manifold necessary for the quantum chemical MP2 and coupled-cluster methods. The space of the occupied orbitals from the HF calculation is projected out from the PGTOs, ensuring that they solely span the virtual space. The rediagonalization of the Fock matrix in this newly constructed virtual space allows for a canonical formulation of quantum chemical techniques. This enables considerably fewer states to be involved in many-body calculations.^54^ The method to obtain PGTOs invokes a pseudization procedure of the sharply peaked Gaussian basis sets, which follows the work of Kresse and Hafner*.*^55^ A more detailed explanation of PGTOs and their application to periodic systems is given in Ref. 54. PGTOs allow for a controllable and reliable extrapolation of the adsorption energies to complete basis set limit results. For the present calculations, Dunning’s contracted aug-cc-pVDZ (AVDZ), aug-cc-pVTZ (AVTZ), and aug-cc-pVQZ without *g* functions (AVQZ–*g*) basis sets^56,57^ were pseudized and expanded in a plane-wave basis set.^54^ Augmented functions were not included for the Li atom because they possess a small exponent for the radial part that introduces linear dependencies in the virtual orbital space. The AVQZ–*g* basis set used here does not encompass *g* angular momentum functions since the corresponding pseudization procedure has not yet been implemented in vasp. Counterpoise corrections (CPs) to the basis set superposition error (BSSE)^58^ were included in all correlated quantum-chemical calculations with plane-waves that employ PGTOs for the virtual states.

Canonical periodic MP2 calculations using PGTOs were performed with the vasp code.^14,18^ The evaluation of the two-electron-four-index integrals requires the intermediate Fourier-transformed overlap densities which are expanded into an auxiliary plane-wave basis.^18^ The kinetic energy cutoff $E_{\chi }$ defining this auxiliary basis set was set to 200 eV. All reported MP2 adsorption energies have been checked for convergence with respect to this cutoff. Table I shows the convergence of the MP2 adsorption energy with respect to the cutoff energy.

**TABLE I.** MP2 adsorption energy against the cutoff energy $E_{\chi }$ of the auxiliary basis set. One-particle states were expanded in a plane-wave basis set with a cutoff of 500 eV, while the virtual states were constructed using an AVTZ basis set.

$E_{\chi }$ (eV)	*E*_ads_ (meV)
50	242
100	214
150	211
200	211
250	211
300	211

Periodic CCSD calculations were performed using the two-electron-four-index integrals calculated within the PAW method in vasp. To further reduce the computational cost of coupled cluster methods, we first minimize the number of virtual orbitals. Pseudized Gaussian orbitals were placed only on the top-most layer of the LiH slab. In a second step, the auxiliary plane-wave basis, required for the evaluation of the Coulomb integrals, employed a kinetic energy cutoff of 100 eV. MP2 calculations reveal that this approximation yields adsorption energies that deviate by 3 meV from those obtained using a cutoff of 200 eV as indicated in Table I.

Kats and Manby^59^ proposed an approximation to CCSD theory that neglects exchange processes between different clusters which is formally still exact for two-electron systems. The resultant theories have been called distinguishable cluster theories because they violate the indistinguishability of electrons in a many-electron system. However, it has been shown that distinguishable cluster approximations such as distinguishable cluster singles and doubles (DCSD) correctly dissociate a number of diatomic molecules and yield very accurate equilibrium geometries and interaction energies for many molecular systems, outperforming the accuracy of CCSD theory at the same computational cost.^60–62^ Motivated by these findings, we also performed periodic DCSD calculations for the adsorption energy.

Finally, a *δ*CCSD(T) correction was applied as the difference between canonical periodic CCSD(T) and MP2 calculations using the AVDZ PGTOs (placed in the top-most layer) and an H_2_O + Li_8_H_8_ simulation cell.

### C. Gaussian-basis calculations

The Gaussian-type-orbital-based HF calculations were performed with the crystal program package.^63^ To this end, a 64-atom supercell, a 3×3×1 *k*-mesh, and tightened integral prescreening thresholds (TOLINTEG 8 8 8 25 100) were employed. A valence-triple-zeta (VTZ) basis set combining Ahlrichs’ functions for low angular momentum^64,65^ and Dunning’s cc-pVTZ basis set for high angular momentum orbitals was used for the H and O atoms. The Li atoms were described by an optimized basis set already available from previous calculations on the LiH crystal^22^ (basis set A). The local MP2 (LMP2) and the explicitly correlated local MP2 (LMP2-F12)^66^ calculations were performed with the cryscor code. For these calculations, the VTZ basis set was augmented by additional diffuse orbitals using the dual basis set technique^67^ leading to AVTZ quality. For the O and H atoms, these were the *d* and *f* (*p* and *d* for H) orbitals from the aug-cc-pVTZ basis set, and for Li, these were the *s*, *p*, *d*, and *f* orbitals of the basis set B of Ref. 22. The effect of the augmented orbitals on the HF energy was estimated via the first order singles.^67^

The correlation energy was calculated in the direct space, considering H_2_O–LiH inter-pairs with inter-orbital separation up to 15 Å. From 15 Å to infinity, the pair-wise R^−6^ extrapolation was employed.^17^ For the LiH intra-pairs, the (converged) value of 6 Å was used as the inter-orbital cutoff distance. In the evaluation of the local F12 correction (within the 3*A approximation^68^), which is of much shorter range than LMP2 itself,^66^ the pair cutoff distances were reduced to 4 Å and 8 Å for the LiH intra-pairs and water–LiH inter-pairs, respectively.

The pair-specific truncated virtual spaces of each Wannier function (WF) pair in the projected atomic orbital (PAO)-based LMP2 is constructed as the union of the two related orbital domains. In our calculations, the latter comprised, for each LiH WF, the PAOs on the H atom and the five nearest neighbour Li atoms. The orbital domains of the WF located on water comprises all three water atoms. The same domains were also employed for the local resolution of identity (RI) domains^66^ in the LMP2-F12 calculations. For the density fitting of the electron repulsion integrals and the local RI approximation of the F12 method, the auxiliary basis sets of Weigend and co-workers^69,70^ were used, i.e., aug-cc-pVTZ-mp2fit and cc-pVTZ-jkfit, respectively.

In the periodic LMP2 and LMP2-F12 calculations, the 1*s*^2^ core states of O and Li were kept frozen. Nevertheless, the correlated core contribution of the 1*s*^2^ states of the Li atoms was computed at the MP2 level with an aug-cc-pwCVTZ basis set on the H_2_O + Li_25_H_25_ cluster using the molpro program package.^71^ The core-correlation contribution to the interaction is relatively short-range making further expansion of the cluster not necessary. Moreover, coupled-cluster calculations on finite clusters were also performed using the molpro code.

### D. DMC calculations

DMC calculations have been performed with the Casino code,^72^ using Dirac–Fock pseudo-potentials (PP)^73^ and trial wave functions of the Slater–Jastrow type,

$$\Psi_{T}\left(R\right)=D^{↑}D^{↓}e^{J},$$ (2)

where $D^{↑}$ and $D^{↓}$ are Slater determinants of up- and down-spin single-electron orbitals, respectively, and *e*^*J*^ is the so-called Jastrow factor, which is the exponential of a sum of one-body (electron-nucleus), two-body (electron-electron), and three-body (electron-electron-nucleus) terms. The parameters in the Jastrow factor were optimised by minimising the variance of the variational Monte Carlo energy, which for the system with one water molecule on a two-layer 3×3 LiH surface supercell was reduced to just over 1 Ha^2^ (740 eV^2^).

The imaginary time evolution of the Schrödinger equation has been performed with the usual short time approximation, using the locality approximation^74^ to treat the non-local part of the pseudopotentials.

The single particle orbitals have been obtained by DFT plane-wave calculations using the local density approximation and a plane-wave cutoff of 3400 eV, using the pwscf package,^75^ and re-expanded in terms of B-splines,^76^ using the natural B-spline grid spacing given by $a=\pi ∕G_{\text{max}}$, where *G*_max_ is the length of the largest vector employed in the plane-wave calculations.

The DMC calculations were then performed with no periodic boundary conditions in the direction perpendicular to the surface, using the Ewald interaction to model electron-electron interactions. DMC adsorption energies were computed as follows:

$$E_{\text{ads}}=E_{s}-E_{b},$$ (3)

where *E*_*b*_ is the energy of the system with the water molecule at its equilibrium position on the surface and *E*_*s*_ is the energy of the system in which the water molecule has been displaced vertically by 10 Å, without relaxing its structure. In the latter configuration, the residual interaction energy between the molecule and the surface is negligible, and this definition of *E*_ads_ maximises DMC cancellation of time step errors.^44,77^

Adsorption energies were calculated using time steps between 0.001 and 0.05 a.u., and we found that with a time step of 0.02 a.u. *E*_ads_ is converged to better than 10 meV.

## III. RESULTS

In order to assess the accuracy of different theories and computational procedures, we study the adsorption of a single water molecule on the (001) surface of LiH. We present the results of DFT calculations, different periodic MP2 and coupled-cluster techniques, and compare these methods with DMC. We first discuss the convergence studies of the various theories with respect to the basis set, finite-size effects, and the number of LiH slabs, and then we compare the adsorption energies of the different methods.

### A. Finite-size and basis set convergence

The finite-size and the basis set convergence studies summarized in this section employ a 2-layer LiH substrate as shown in Fig. 1.

We first discuss the convergence of the DFT-PBE and HF adsorption energies with respect to the system size. DFT-PBE and HF results using different implementations are summarized in Table II. The converged results are in excellent agreement using plane-waves and Gaussian basis sets, with vasp and crystal, respectively. DFT-PBE results are converged already with a 32-atom LiH surface slab due to the inability of DFT-PBE to describe long-range dispersive interactions. HF results also exhibit a very fast rate of convergence albeit underestimating the adsorption energy compared to DFT-PBE significantly due to the neglect of any electronic correlation effects.

**TABLE II.** DFT-PBE and HF adsorption energies for water on 2-layer LiH substrates with different number of atoms in the supercell and different *k*-meshes. The reference 2-layer geometry with 64-atoms is shown in Fig. 1. The DFT-PBE and HF calculations have been performed with vasp and employ a 500 eV kinetic energy cutoff. The HF crystal calculations with an AVTZ-quality basis set and a 3×3×1 *k*-mesh yield a value of 14 meV.

*E*_ads_ (meV)			
*k*-mesh	Atoms	PBE	HF
(Γ-point)	32	219	10
(Γ-point)	64	215	14
(Γ-point)	128	215	15
(3×3×1)	64	214	15

We now turn to the discussion of the adsorption energies using different implementations of MP2 theory. LMP2-F12 is expected to provide results very close to the basis set limit and, with the settings given in Sec. II C, also very close to the thermodynamic limit. It yields an adsorption energy of 238 meV. The latter value consists of 14 meV of HF, 189 of the frozen-core periodic LMP2/AVTZ, 18 meV of the F12 correction, and 17 meV of the core contribution. Using the basis set correction from the LMP2-pF12 approach, which is an approximation to LMP2-F12,^79^ leads to a similar value of 235 meV.

**FIG. 2.**
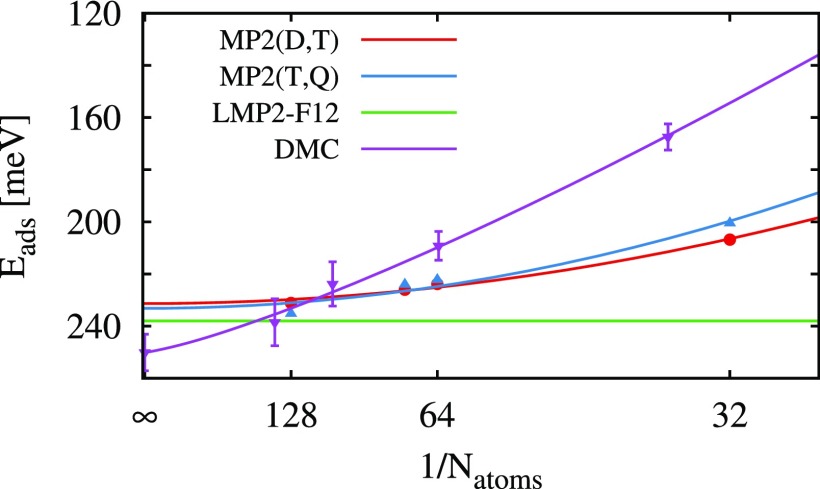
Dependence of the adsorption energy *E*_ads_ of H_2_O on LiH on the number of atoms of the substrate at different levels of theory and basis set extrapolations. The fitted lines correspond to 1/*N*^2^ for the MP2 energies and 1/*N*^5/4^ for the DMC energies. MP2 results employ AV(D,T)Z and AV(T,Q)Z basis set extrapolations.^80^ The LMP2-F12 result corresponds to the thermodynamic limit. On the *x*-axis, N_atoms_ is indicated instead of 1/N_atoms_.

Canonical MP2 energies need to be converged with respect to both the basis set size and to the LiH surface size. Table III summarizes canonical MP2 adsorption energies obtained for varying basis set and supercell sizes. AV(D,T)Z and AV(T,Q–*g*)Z extrapolated adsorption energies agree to within 2–6 meV for all studied system sizes. We note that the AV(T,Q–*g*)Z extrapolation is somewhat less reliable due to the absence of *g* angular momentum functions in the AVQZ values. We find that the MP2 adsorption energies converge as 1/*N*^2^, where *N* denotes the number of atoms in the LiH substrate. This behaviour is expected from the long-range decay of pairwise van der Waals contributions in two-dimensional systems. The convergence of the finite-size effects for the various basis set extrapolated MP2 results can be seen in Fig. 2. Using the 1/*N*^2^ behaviour, we can extrapolate the MP2 adsorption energies to the thermodynamic limit (N→∞), yielding 231 meV and 233 meV for AV(D,T)Z and AV(T,Q–*g*)Z, respectively. The 5 − 7 meV difference between the canonical MP2 and LMP2-F12 is likely due to the remaining basis set incompleteness in the correlation energy of the former method. Notwithstanding, the agreement of the two different schemes, which have very little in common, is impressive. The F12-based explicit correlation techniques combined with local approximation schemes accelerate the convergence of the MP2 correlation energy. Its close agreement with the periodic canonical results suggests that PGTOs provide an adequate virtual basis set for correlated calculations in plane-waves.

**TABLE III.** Canonical MP2 adsorption energies for water on 2-layer LiH substrates with different number of atoms in the computational supercell. The calculations were performed with vasp and employ PGTOs for the virtual orbitals alongside the Γ-point approximation. The thermodynamic limit is obtained from a 1/*N*^2^ extrapolation (*N* denotes the number of atoms in the LiH substrate). The LMP2-F12 and LMP2-pF12 adsorption energies are 238 and 235 meV, respectively.

$E_{\text{ads}}^{\text{MP}2}$ (meV)					
Atoms	AVDZ	AVTZ	AVQZ–*g*	AV(D,T)Z	AV(T,Q–*g*)Z
32	162	193	198	207	201
64	181	211	218	224	222
72	185	213	220	226	224
128	188	218	228	231	235
∞	189	219	227	231	233

DMC adsorption energies^47^ against the number of atoms in the simulation supercell are provided in Table IV. The DMC adsorption energy converges more slowly with respect to the supercell size than the MP2 energy as shown in Fig. 2, due to the long ranged nature of the real-space exchange-correlation hole and reduced screening in lower dimensional materials. Drummond *et al.* proposed a 1/*N*^5/4^ extrapolation for the two-dimensional systems.^78^ Despite its statistical uncertainty, the thermodynamic limit of the DMC adsorption energy suggests that the MP2 error for this system is small but not negligible and thus a higher-order quantum chemical treatment is desirable.

**TABLE IV.** DMC adsorption energies for water on 2-layer LiH substrates with different number of atoms in the computational supercell.^47^ The thermodynamic limit is obtained from a 1/*N*^5/4^ extrapolation.^78^

$E_{\text{ads}}^{\text{DMC}}$ (meV)	
Atoms	CBS
36	167 (5)
64	209 (5)
100	224 (8)
144	239 (9)
∞	250 (7)

**FIG. 3.**
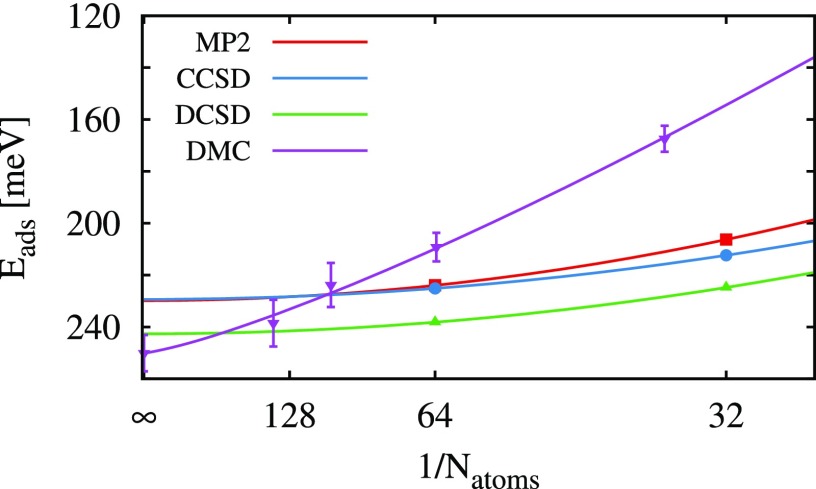
Adsorption energy *E*_ads_ of H_2_O on LiH for different supercell sizes and levels of theory. Coupled-cluster and MP2 calculations were done using PGTOs only on the top-most layer of the LiH substrate. The fitted lines correspond to 1/*N*^2^ for the coupled-cluster and MP2 energies and 1/*N*^5/4^ for the DMC energies. The coupled-cluster and MP2 results employ AV(D,T)Z basis set extrapolation.^80^ On the *x*-axis, N_atoms_ is indicated instead of 1/N_atoms_.

Periodic coupled-cluster calculations were performed with PGTOs for the virtual orbitals. However, these Gaussian-type functions were placed only on the top-most layer of the LiH surface to reduce the computational cost. Additionally, only supercells with 32 and 64 atoms were used to model the LiH slab. AVDZ and AVTZ Gaussian basis sets were used for the construction of the PGTOs, and all results are extrapolated with respect to the basis set and the number of atoms in the supercell. MP2 results utilizing Gaussian orbitals for the full LiH surface and a finite-size extrapolation using four points verify that correlation effects are captured adequately via only top-most layer virtual states and a finite-size extrapolation using two points. The error of this simplification is about 1 meV in the MP2 energy. Consequently, it is reasonable to assume that coupled-cluster results obtained using the same simplification provide a similarly converged estimate. MP2 and coupled-cluster results are summarized in Table V and Fig. 3. The CCSD adsorption energies are close to those of MP2, differing only by 1 meV. However, the extrapolated DCSD results deviate quite significantly from the CCSD and MP2 results, yielding an adsorption energy of 243 meV in better agreement with the DMC values.

**TABLE V.** MP2 and coupled-cluster adsorption energies using LiH substrates with different number of atoms in the supercell. PGTOs were used for the virtual orbitals in the top-most layer of the LiH surface. The thermodynamic limit is obtained via a 1/*N*^2^ extrapolation.

$E_{\text{ads}}^{\text{MP2}}$ (meV)			
Atoms	AVDZ	AVTZ	AV(D,T)Z
32	157	192	207
64	173	209	224
∞	180	216	230

$E_{\text{ads}}^{\text{CCSD}}$ (meV)			
Atoms	AVDZ	AVTZ	AV(D,T)Z
32	152	195	212
64	172	209	225
∞	180	215	229

$E_{\text{ads}}^{\text{DCSD}}$ (meV)			
Atoms	AVDZ	AVTZ	AV(D,T)Z
32	162	206	225
64	183	222	238
∞	192	229	243

Finally, a *δ*CCSD(T) correction scheme was applied to both the canonical and the local MP2 results. In the former case, the correction *δ*CCSD(T) was defined as

$$E^{\delta \text{CCSD(T)}}=E_{{\text{H}}_{2}{\text{O+(LiH)}}_{\infty }}^{\text{MP2}}+E_{{\text{H}}_{2}{\text{O+Li}}_{8}{\text{H}}_{8}}^{\text{CCSD(T)}}-E_{{\text{H}}_{2}{\text{O+Li}}_{8}{\text{H}}_{8}}^{\text{MP2}}\;,$$ (4)

where canonical CCSD(T) and MP2 calculations were performed using an H_2_O + Li_8_H_8_ 2-layer supercell (with an identical orientation of the water molecule as for the larger supercells) and an AVDZ basis set in a plane-wave representation. $E_{{\text{H}}_{2}{\text{O+(LiH)}}_{\infty }}^{\text{MP2}}$ is the thermodynamic limit of the MP2 adsorption energy using the AVD(T,Q–*g*)Z basis set extrapolation. This yields an adsorption energy of 254 meV.

The *δ*CCSD(T) corrections to the LMP2-F12 results were computed using finite clusters. In this case, the canonical MP2 and CCSD(T) adsorption energy calculations were done on an H_2_O + Li_9_H_9_ 2-layer finite cluster using the AV(D,T)Z basis sets. The water molecule geometry was taken from the periodic supercells. The correction *δ*CCSD(T) for the periodic system was defined as

$$E^{\delta \text{CCSD(T)}}=E_{{\text{H}}_{2}{\text{O+(LiH)}}_{\infty }}^{\text{LMP2-F12}}+E_{{\text{H}}_{2}{\text{O+Li}}_{9}{\text{H}}_{9}}^{\text{CCSD(T)}}-E_{{\text{H}}_{2}{\text{O+Li}}_{9}{\text{H}}_{9}}^{\text{MP2}}\;,$$ (5)

yielding an adsorption energy of 256 meV. Incidentally we note that one cannot construct a periodic Li_9_H_9_ supercell and therefore a Li_8_H_8_ slab was used for the plane-wave based *δ*CCSD(T). Furthermore, the finite-size error of the correction was estimated as the difference between local LCCSD(T0)—LCCD[S]-R^−6^ calculations^81–83^ on H_2_O + Li_9_H_9_ and H_2_O + Li_25_H_25_ clusters. This difference turned out to be of the order of 0.3 meV. However, we note that a *δ*CCSD correction, defined in an analogous way as *δ*CCSD(T), provides an adsorption energy of 219 meV, which deviates somewhat from the periodic CCSD result. In contrast, a periodic *δ*CCSD correction, defined in an analogous way as *δ*CCSD(T), yields an adsorption energy of 227 meV, very close to the canonical CCSD result. Thus the finite-cluster *δ* approach might still contain a certain error.

### B. Convergence of the adsorption energy with the number of layers

In this section, we investigate the adequacy of the chosen slab model, which consists of just two LiH layers, for studying the adsorption of water. Generally, the convergence of the adsorption energy with the number of layers in the slab is expected to be governed by long-range effects, such as electrostatics (attractive or repulsive) and dispersion (attractive). Importantly, electrostatics are already captured at the DFT or HF levels, while dispersion is not (unless the dispersion correction is added or a special DFT functional is used, which is able to describe dispersion).

Table VI demonstrates by how much the adsorption energy grows or declines if further layers are added to the slab, as computed by DFT and HF. In order to isolate the dispersion contribution, we provide the -D3 contribution separately, as well as the LMP2 correlation energy. For dispersion alone, it is actually possible to obtain convergence with the number of layers: -D3 is very inexpensive and thus can be computed for very thick slabs, while for LMP2 the inter-adsorbate-slab contribution can be extrapolated to a semi-infinite slab using the pair-specific *C*_6_ coefficients fitted to the actual LMP2 pair energies (see Ref. 84 for details).

**TABLE VI.** Convergence of the adsorption energy (DFT-PBE, HF), the dispersion correction (-D3), and the correlation energy (LMP2) with respect to the slab thickness. The provided energies (in meV) represent the excess or depletion in the energy with respect to the 2-layer slab model due to additional layers. All the calculations employed the 4×4 surface supercell. The ∞ symbol indicates the converged D3 and LMP2 value. The latter is obtained by extrapolation of the inter-LiH-Water energy from the 3-layer model to a semi-infinite slab by means of the slab replication technique of Ref. 84, employing pair-specific *C*_6_ coefficients fitted to the actual LMP2 pair energies. The result of such an extrapolation from the 2-layer model is given in parentheses.

No. of layers	PBE	HF	-D3	LMP2
3	−0.15	−1.51	+5.36	+2.44
4	−0.16		+7.01	
∞			+8.44	+4.66(+4.97)


The PBE and HF results suggest that for the non-dispersive contributions, the two-layer slab is already an adequate model. Dispersion, on the contrary, is not entirely converged with just two LiH-layers. However, at the scale of the whole adsorption energy, the lack of a few meV of dispersion in the two-layer model can be tolerated.

### C. Comparison of methods

**FIG. 4.**
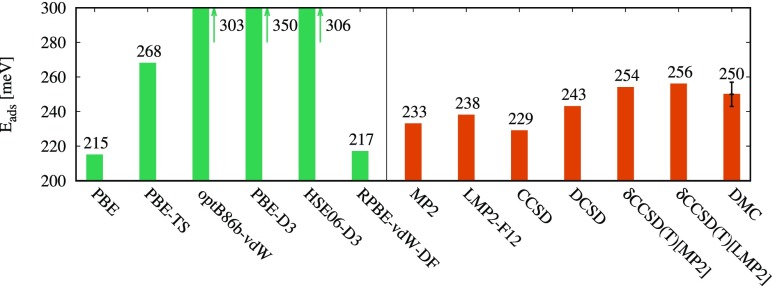
Converged adsorption energies of a water molecule on the LiH surface at different levels of theory. PBE and several van der Waals functionals shown on the left. Wave function based methods ranging from MP2 to *δ*CCSD(T) and DMC shown on the right.

We now summarize the converged adsorption energies and compare them to a small set of widely used density-functionals. All reported results employ a 2-layer LiH substrate as in Fig. 1. We believe that the mutually agreeing DMC and *δ*CCSD(T) results can be considered as the most reliable benchmark for the present system, yielding adsorption energies between 250 (±7) meV and 256 meV. For comparison, the adsorption energy of each method is depicted in Fig. 4. A sizeable variation in the adsorption energies is evident between different van der Waals functionals (PBE-TS,^49^ optB86b-vdW,^53^ PBE-D3,^48^ HSE06-D3,^85^ RPBE-vdW-DF^50^), as well as PBE. The PBE functional underestimates the adsorption energy by roughly 45 meV, in a large part due to its lack of dispersive interactions. Grimme’s D3 correction^48^ accounts for such interactions, albeit overestimating the adsorption energy for the current system, predicting a PBE-D3 adsorption energy of 350 meV, consistent with similar findings for water adsorption on ionic surfaces.^86^ We note that this overestimation is less pronounced when the HSE06^87,88^ hybrid functional is used in conjunction with D3, yielding a value of 306 meV. This can partly be attributed to the fact that the HSE06 functional underestimates the adsorption energy compared to PBE by as much as 85 meV. The optB86b-vdW^53^ results also overbind the water molecule by roughly 45 meV, while the RPBE-vdW-DF^50^ adsorption energy exhibits a similar underbinding as for the case of PBE. The best van der Waals functional estimate is provided by the Tkatchenko and Scheffler functional (PBE-TS) with iterative Hirshfeld partitioning.^89,90^ The latter yields an adsorption energy of 268 meV in good agreement with *δ*CCSD(T) results. These results illustrate the difficulties in van der Waals functionals. The PBE functional is known to provide non-electrostatic binding between closed shell systems. This attraction is rather an artifact than a real dispersive interaction. At the same time, this artificial attraction provides a quantitatively reasonable effective substitute for dispersion. However, if the physically correct dispersion is added on top, it becomes difficult to avoid double counting, leading to a deterioration of the quantitative accuracy.

Figure 4 also shows the various wave function estimates of the adsorption energy. Canonical MP2 theory underestimates the adsorption energy by 17 meV compared to DMC, while LMP2-F12 provides a slightly better estimate, partly due to the explicit correlation, leading to an improved convergence with respect to the basis set size. The LMP2-F12 adsorption energy is 238 meV, only 11 meV below the DMC result. CCSD constitutes no improvement over MP2 theory for the present case, yielding a binding energy of 229 meV only. The DCSD approximation,^59^ consistent with findings in molecular systems,^59–61^ considerably improves the description of water adsorption on LiH, predicting an adsorption energy of 243 meV, which is within the stochastic error of DMC but still underbinding compared to the triples corrected *δ*CCSD(T) results. In summary, we find excellent agreement between high-level quantum chemistry and QMC techniques as well as between different methods to compute MP2 adsorption energies. Furthermore the correlated wave function based methods yield estimates for the binding energy that lie in a relatively narrow energy window ranging from 229 meV to 256 meV.

## IV. CONCLUSIONS

We have presented a comprehensive comparison between different electronic structure methods including wave function based theories and a small selection of density-functionals for the prediction of the adsorption energy of a single water molecule on the (001) LiH surface.

Quantum chemical methods are systematically improvable, hence yielding increasingly accurate adsorption energies as one moves up the hierarchy to higher orders of theory. Distinguishable cluster theory and inclusion of triple excitations to CCSD theory give the best agreement with the DMC results. We find that MP2 and CCSD reach a similar level of accuracy for this system, slightly underbinding the water molecule on the LiH surface by roughly 20 meV. We also find good agreement between periodic canonical and local implementations utilizing explicit correlation techniques for improved basis set convergence. All these demonstrate that quantum chemical approaches are becoming a robust and reliable tool for condensed phase electronic structure calculations.

We have also employed van der Waals functionals for the study of the same system, finding that these functionals yield a significantly larger spread of adsorption energy estimates compared to the employed many-electron theories. The underestimation and overestimation compared to DMC and *δ*CCSD(T) are as large as 30 meV (RPBE-vdW-DF) and 100 meV (PBE-D3), respectively. Although the PBE-TS functional achieves good agreement with the DMC and *δ*CCSD(T) estimates for the present case, it remains difficult to achieve such a high level of accuracy for a wide class of materials using van der Waals functionals. This study contributes another benchmark system to the literature that can be used to further improve upon the currently available and computationally very efficient van der Waals functionals for cases where higher accuracy is needed.

## SUPPLEMENTARY MATERIAL

See supplementary material for the structure of water adsorption on the (001) LiH surface.
